# Parental and offspring larval diets interact to influence life-history traits and infection with dengue virus in *Aedes aegypti*

**DOI:** 10.1098/rsos.180539

**Published:** 2018-07-18

**Authors:** Kylie Zirbel, Bradley Eastmond, Barry W. Alto

**Affiliations:** Florida Medical Entomology Laboratory, Entomology and Nematology Department, Institute of Food and Agricultural Sciences, University of Florida, Vero Beach, FL 32962, USA

**Keywords:** *Aedes aegypti*, parental effects, dengue virus infection, life-history traits, larval nutrition

## Abstract

The environmental conditions experienced by parents can influence offspring phenotype along with the conditions experienced by offspring. These parental effects are clear in organisms that display parental care and are less clear in other organisms. Here, we consider effects of parental and offspring larval nutrition on offspring development time, survivorship and infection with dengue virus in *Aedes aegypti*, the mosquito vector of dengue, chikungunya, yellow fever and Zika. Parents were raised on either high or low larval detritus inputs with subsequent offspring being divided into two groups, one receiving high nutrients and the other low. Low nutrient females from low nutrient parents (LL) developed significantly slower than those from high nutrient parents (HL). Females from all parent by offspring nutrient treatment groups were equally likely to become infected with dengue virus at 24 h, 3 days and 14 days. After 14 days, high nutrient females from low nutrient parents (LH) had 11 times higher viral titres and more disseminated infections than high nutrient females from high nutrient parents (HH). These results suggest that carry-over environmental stress from the parental generation can influence life histories and arbovirus infection in *Ae. aegypti* females. We found males to be robust to the life-history parameters measured, suggesting sex-specific differences which may relate to their lower nutrient requirements for metamorphosis.

## 1. Introduction

Parental effects occur when the environmental conditions experienced by parents influence offspring phenotypes. These effects can be considered a type of inherited phenotypic plasticity [1] and their importance is clear in organisms displaying parental care [2]. However, parental effects influence the ecology and evolution of a wide range of taxa [2–4]. Adaptive parental effects occur when environmental conditions experienced by parents enhance offspring fitness [5]. Maladaptive parental effects occur when environmental conditions result in poorer quality offspring [6]. The conditions leading to parental effects vary across species and traits considered. In insects, parental nutrition has been linked to parental effects in several orders [7–11]. One offspring trait that has been linked to parental effects is immunity [9,11,12]. These studies demonstrate the importance of considering parental nutrition effects on immunity in other systems.

Container mosquitoes have received considerable attention in ecological studies partially because they vector arboviruses including dengue, chikungunya, yellow fever and Zika. Dengue alone is estimated to infect 390 million people per year, with 96 million cases with clinical manifestations [13]. The term ‘container’ refers to the natural and artificial larval habitats these mosquitoes rely upon. Allochthonous detritus inputs to these containers, including plant and animal detritus, serve as basal resources for microorganisms upon which developing larvae feed. The quantity and type of detritus in a container affect larval growth and development as does competition between conspecifics and heterospecifics [14]. Nutrient limitation is common in container systems [15].

In mosquitoes, larval nutrient limitation is associated with reduced survival to adulthood, longer development, compromised growth and reduced adult fitness (e.g. [11–17]). Nutritionally deprived females often lay fewer eggs [16] and have reduced fecundity [18]. Larval nutrition is positively associated with body size, a measure of net growth and metabolic reserves [18]. During nutritive stress, teneral protein, lipid and carbohydrate reserves acquired during larval development can be mobilized to promote survival [18,19]. Larger adult mosquitoes generally live longer [20,21], take larger blood meals [22] and have higher total fecundity [18] than smaller mosquitoes. However, after an initial blood meal, survival may no longer be associated with size [23]. Body size, adult longevity and blood-feeding behaviour are associated with vector potential [19,20,24]. *Aedes aegypti* and *Anopheles gambiae* often take multiple blood meals per gonotrophic event, a behaviour associated with the high transmission of pathogens by these species [24]. The ability of the vector to survive the extrinsic incubation period (EIP) of a pathogen (period of development in the vector) and to then engage in subsequent blood feeding is necessary for transmission.

Larval nutrition within a generation has been shown to influence vector competence of mosquitoes in complex ways. Vector competence refers to susceptibility to infection, replication and transmission of pathogens. Larval nutrient limitation is associated with increased vector competence in *Ae. albopictus* [25], *Ae. triseriatus* [26] and *Culex tritaeniorhynchus* [27]; both increased [17] and decreased vector competence in *Ae. aegypti* [28]; and no effect on vector competence in *C. tarsalis* [29]. The influence of larval nutrition on vector competence is associated with barriers to infection and innate immunity [26,30]. Larval nutrition may have epidemiological consequences because physical and physiological barriers to infection influence viral dissemination to secondary tissues, including mosquito saliva necessary for transmission. Given the importance of larval nutrition in influencing adult mosquito phenotypes and fecundity, a logical step is to consider how larval nutrition experienced by parents influences subsequent offspring.

The relationship between larval nutrition, immunity and parental effects may have consequences for mosquitoes that transmit pathogens. In the mosquito *An. stephensi*, parental food during the larval stage did not significantly affect offspring emergence time, size or survival but did influence offspring fecundity [8]. In *An. gambiae*, daughters of females reared in nutrient-deprived conditions as larvae were more likely to be infected with the malaria parasite, *Plasmodium berghei* [31]. However, maternal infection with a microsporidian, in *An. gambiae*, resulted in reduced susceptibility to malaria in daughters possibly due to immune priming [31]. These studies demonstrate that interactions between the parental environment and offspring phenotype are complex. Parental effects can influence host–parasite interactions in important vector species and consequently should be considered in research [32], especially for emerging arboviruses, which remain under-investigated.

In this study, we tested the hypothesis that parental and offspring larval nutrition affects larval and adult characteristics in offspring that have epidemiological consequences for the transmission of arboviruses. From this hypothesis, and based upon prior work on the effects of larval environment on mosquito vector biology, we predict that offspring from parents reared in low nutrients will have decreased fitness correlates and greater susceptibility to dengue infection relative to offspring from parents reared in high nutrient larval conditions. We also predict that high nutrient offspring will have increased fitness correlates and lower susceptibility to infection relative to low nutrient offspring regardless of parental nutrition. We predict that interactive effects of parental and offspring nutrition will result in high food offspring from high food parents having increased fitness correlates and lower susceptibility to infection relative to other treatment combinations. Here, we investigate whether larval nutrition experienced by parents and offspring in *Ae. aegypti* influences offspring (i) life-history traits (growth, development, survival to adulthood) and (ii) susceptibility to dengue-1 virus infection. Though we focus on *Ae. aegypti* and dengue, due to the importance of this mosquito as a vector and the global burden of dengue, the concepts improve our general understanding of the potential role of parental effects on arbovirus mosquito vectors.

## 2. Material and methods

### 2.1. Source and rearing of parental mosquitoes

*Aedes aegypti* used were from an established colony of wild-caught mosquito larvae collected from Key West, FL, in 2012. We used *Ae. aegypti* from Key West as this population was responsible for dengue-1 virus transmission in 2009 and 2010 [33]. Insectary conditions were set to 28 ± 0.5°C and a 12 L : 12 D photoperiod and sustained throughout the experiment. Colony mosquito larvae were reared in enamel pans (24 × 36 × 5 cm) in 1.5 l of water in cohorts of approximately 200 mosquitoes on an equal mixture of brewer's yeast and lactalbumin given in 0.2 g increments two to three times per week. Pupae were transferred to water-filled cups and placed into plastic cages (45.7 × 45.7 × 45.7 cm, BugDorm, MegaView Science Co. Ltd, Taichung, Taiwan) to emerge as adults. Adults were maintained on a 20% sucrose solution from cotton wicks and blood fed on live chickens approximately once per week. Chicken care followed the animal use and care policies of the University of Florida's Institutional Animal Care and Use Committee (IACUC Protocol 201003892).

To reduce the possibility of parental effects generated in the wild, the F_4_ generation was used as the parental (P) generation for this study [34]. P larval nutrition treatments included natural sources of plant and invertebrate detritus and microorganisms. Ten 2.0 l cylindrical plastic containers (15.5 cm × 17.1 cm, height × diameter) for each treatment group were used to rear the P larvae. Each container received 2.0 l of tap water and 10 ml of tyre water inoculum (collected from tyres in at the Florida Medical Entomology Laboratory (FMEL) campus) with associated microorganisms. Each container received senescent live oak leaves (*Quercus virginiana*), a predominant host tree for container habitats in Florida and dead field crickets (*Gryllus* sp.) in amounts varying by treatment (low nutrition, 4 g oak leaves + 0.06 g crickets; high nutrition, 12 g oak leaves + 0.2 g crickets). The oak leaves were collected from the FMEL campus and dried for 24 h at 70°C. The crickets were obtained from the local pet supply store and dried for 48 h at 60°C using established methods [35].

Contents were incubated in containers for 4 days prior to use to allow microbial populations to establish. P generation eggs were hatched in containers with 1.0 l tap water and 0.2 g of an equal mixture of brewer's yeast and lactalbumin. After 24 h, larvae were removed from nutrients, rinsed and 200 were added to each experimental container (0.1 larvae ml^−1^). Larval density approximated the mean natural density in Florida containers occupied by *Ae. aegypti* and competitor *Ae. albopictus* (*N* = 790, mean ± s.e., 0.17 ± 0.02, range 0.00083–3.08 larvae ml^−1^) [36]. Supplemental food consisting of half the initial amount was added every 7 days. P larvae were checked daily for pupation. Pupae were transferred to plastic vials containing water and sealed with cotton until emergence as adults. As adults, mosquitoes were sexed and placed in treatment-specific cages containing a water-filled cup lined with paper towel for an oviposition substrate. No more than 50 mosquitoes were added to each cylindrical cardboard cage (9.7 × 9.7 cm, height × diameter). Each cage contained approximately a 1 : 1 male to female ratio, and females within a 3-day age span.

**Figure 1.**
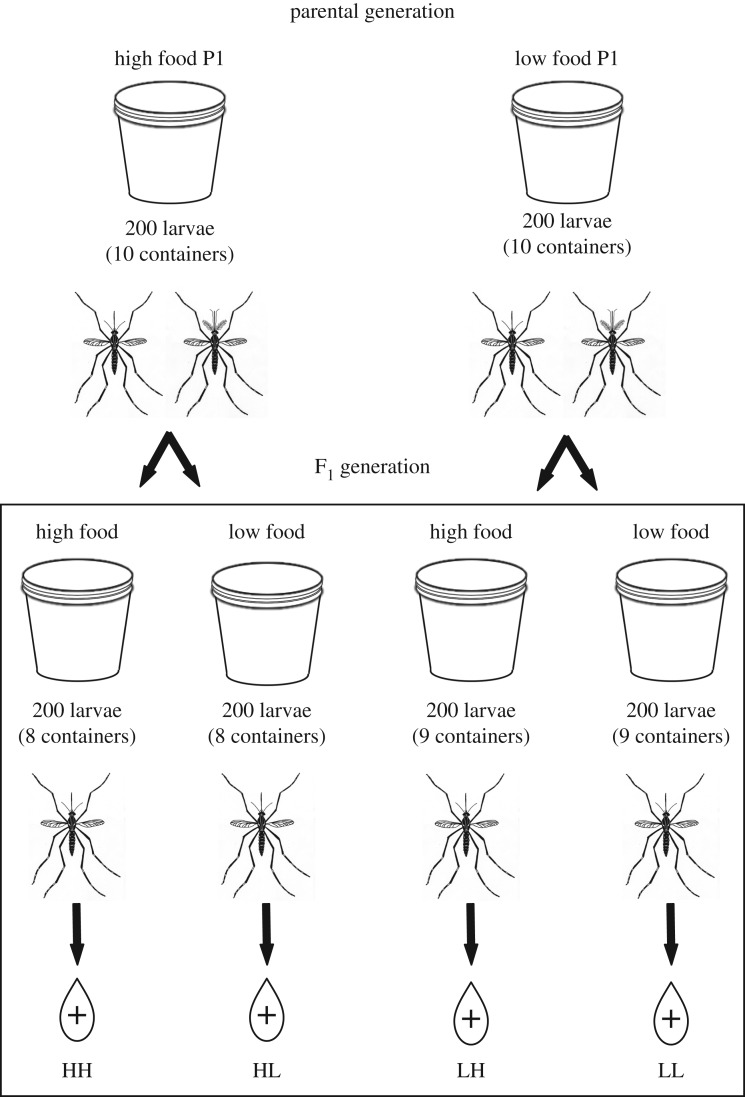
Experimental design for parental and offspring nutrition treatments.

The mosquitoes were given 8–11 days to mate and were sustained on a 20% sucrose solution. After the mating period, sucrose was replaced with water for 2 days to increase propensity to blood feed. Mosquitoes were then offered a bovine blood meal warmed to 37°C using an artificial membrane feeding system (Hemotek^®^). Feeding rates were 73.7% for high food females and 64.0% for low food females. Fully engorged females were held in cages for 7 days during which gravid females laid eggs. Eggs were removed after 7 days and maintained in a humid environment until hatching (see Offspring rearing). Two high food and one low food parental treatment containers were lost due to water quality issues. This resulted in four fewer offspring containers from high food parents and two from low food parents than originally planned (see figure 1 for experimental design).

### 2.2. Offspring rearing

Four days prior to hatching the offspring (F_1_ generation), experimental containers were set up for each F_1_ treatment group. Hatching was staggered to ensure larvae were similar in age. Experimental containers were the same as those for the parental treatment groups. The F_1_ generation eggs from each parental container were hatched and 200 of the resulting larvae were reared in low nutrients and 200 were reared in high nutrients using the same methods as the P generation resulting in four different P × F_1_ larval nutrition treatment groups in a split plot design (figure 1). These treatment groups include: high food parents with high (HH) and low food offspring (HL) and low food parents with high (LH) and low food offspring (LL). The treatment manipulation allows for similar genetic diversity between the offspring treatment groups derived from each parental treatment group.

F_1_ larvae were checked daily for pupation. Pupae were transferred to sealed vials until emergence as adults. Development was measured as the time in days from hatch to adult emergence. Survivorship to adulthood was recorded for each experimental replicate calculated from the initial cohort size. Upon emergence, adults were sexed and transferred into treatment-specific cages using a handheld battery-powered aspirator. Adults were maintained on a 20% sucrose solution for 8–10 days post-emergence. Forty-eight hours prior to blood feeding, the sucrose solution was replaced with water to improve feeding on infectious blood. Females were offered blood infected with dengue-1 virus isolated from Key West, FL, see GenBank: JQ675358.1, using the Hemotek^®^ feeding system described by Alto & Bettinardi [37].

### 2.3. Mosquito infection with dengue virus

Dengue virus was propagated for blood meals following previously established methods [37,38]. Briefly, monolayers of African green monkey kidney (Vero) cells, in tissue culture flasks (175 cm^2^), were inoculated with 250 µl of dengue at a multiplicity of infection of 1.2 followed by 1 h incubation at 37°C in a 5% CO_2_ atmosphere. Post-incubation, 25 ml of media (199 media, 10% fetal bovine serum, 0.2% antimycotic and 0.2% penicillin–streptomycin) was added to each flask. After 7 days, the media (containing dengue) were combined with defibrinated bovine blood (Hemostat, Dixon, CA, USA) in a 1 : 1 ratio.

F_1_ females were offered dengue infectious blood for 1 h. Samples of the infectious blood meal were taken prior to blood feeding to determine dengue titres using quantitative reverse transcriptase–polymerase chain reaction (qRT–PCR). Post-blood-feeding females were cold anaesthetized and fully engorged females were separated and housed in cages. Fed F_1_ females were held individually for either 24 h, 3 or 14 days, where 14 days are the approximate EIP of dengue in *Ae. aegypti* at 28°C [39]. The 24 h, 3- and 14-day time points were specifically chosen because at these time points there is differential expression of Toll pathway-related genes in response to a dengue infectious blood meal [40]. The Toll pathway is one component of the innate immune response of mosquitoes to dengue virus [40,41] and as such can influence susceptibility to infection, viral dissemination and transmission potential. After the incubation period, females were individually stored in 2.0 ml centrifuge tubes at −80°C until assayed to determine infection status, body viral titre and (in 14-day mosquitoes) dissemination status. We did not test for disseminated infections in samples from the 24 h and 3-day time points because dissemination is unlikely at these time points [39].

### 2.4. Determination of infection status

F_1_ blood-fed females were dissected using sterilized forceps to separate the body, legs and wings. Wing length was measured, in millimetres from alula to wing tip, to approximate mosquito body size (reviewed in [42]). Bodies were homogenized in 1.0 ml of TRI Reagent^®^ (Molecular Research Center, Inc., Cincinnati, OH, USA) with three glass beads at 25 Hz for 3 min using a Qiagen^®^ Tissue Lyser. Total RNA was extracted following the TRI Reagent^®^ protocol and stored at −80°C until qRT–PCR assays could be completed. RNA was then extracted from leg tissues of mosquitoes with dengue positive bodies as determined by qRT–PCR. Viral dissemination into the haemocoel was measured at 14 days as indicated by infection of body and leg tissues [43]. Disseminated infection is regarded as a state of advanced infection and a prerequisite for transmission. qRT–PCR for dengue was performed using the SuperScript^®^ III Platinum^®^ one-step qRT–PCR kit (Invitrogen Life Technologies, Carlsbad, CA, USA) and fluorogenic probes (TaqMan^®^, Applied Biosystems, Foster City, CA, USA). Dengue-1-specific primers and probes were designed by Callahan *et al*. [44] and can be found in electronic supplementary material, S1. The PCR reagent quantities and protocol used are described in [45]. Plaque assays, based on methods established in [38], were used to calculate dengue titre in a series of serially diluted standards. RNA from these standards was then extracted and qRT–PCR was used to develop a standard curve in plaque forming unit equivalents (PFUe) ml^−1^. This standard curve was used to quantify viral titre in mosquito tissues.

### 2.5. Statistical analyses

Multi-variate analysis of variance (MANOVA) was used to determine the effect of parental nutrient environment, offspring nutrient environment and parental by offspring nutrient environment on high and low food offspring life-history traits: male development time, female development time and per cent survivorship (PROC GLM, SAS v.9.3). To identify parental larval nutrient effects, the following treatments were compared: HL versus LL and HH versus LH. To consider offspring larval nutrient effects, the following treatments were compared: HH versus HL and LH versus LL. Lastly, to identify parental and offspring combined larval nutrient effects, the following treatments were compared: HH versus LL and LH versus HL. Standardized canonical coefficients were used to measure the relative contribution of each life-history trait to significant treatment effects and their relationship to one another (positive or negative) [46]. In order to satisfy requirements for parametric testing, a natural log transformation was performed on viral titre data. The effect of nutrient treatment (P × F_1_), day and day by nutrient treatment on ln viral titre results were analysed using generalized linear mixed models (PROC GLIMMIX, SAS v. 9.3). Generalized linear mixed models were used because this procedure is more robust to departures from normality than the generalized linear model procedure. Significant effects were further analysed by all pairwise comparisons of nutrient treatment means and adjusted using the Bonferroni correction method [47]. The effect of parental larval nutrition on offspring mosquitoes infected or uninfected as well as absence or presence of disseminated infection was analysed using maximum-likelihood categorical analyses of contingency tables (PROC GENMOD, SAS v. 9.3). The absence or presence of dengue virus in abdomens and leg tissues was determined on an individual female basis with results from each offspring container being pooled together by P × F_1_ nutrient treatment group (HH, HL, LH, LL) due to poor feeding rates in order to meet testing requirements.

## 3. Results

### 3.1. Life-history traits

**Figure 2.**
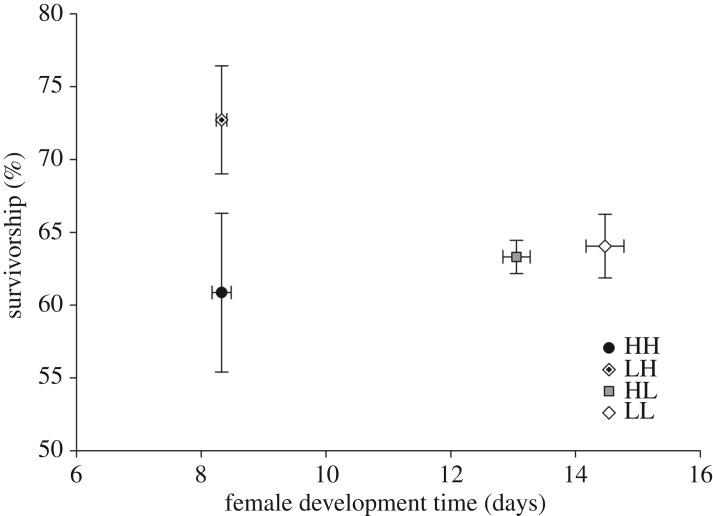
Bivariate plot of least square (LS) means (±s.e.) for offspring female development time and survivorship by treatment.

A total of 4336 offspring emerged to adulthood (2436 males and 1900 females). These adults were used in the life-history trait analysis for development time (males and females) and survivorship. Female size was only estimated for females with measurable wings that successfully fed on blood (511 females). See table 1 for descriptive statistics. MANOVA showed significant parental nutrient effects (*p *= 0.0018), offspring nutrient effects (*p *< 0.0001) and parental by offspring nutrient effects (*p *< 0.0001) on life-history traits (table 2). Multi-variate pairwise contrasts found significant parental effects on offspring when offspring were reared in low nutrients (*p *< 0.0001) but not when offspring were reared in high nutrients (*p *= 0.17). In low nutrient offspring, female development time contributed the most to the significant parental effect followed by survivorship (figure 2). Female size and male development time contributed little to the treatment effect. Low nutrient female offspring from low nutrient parents (LL) developed more slowly (14.47 ± 0.30 days) than low nutrient female offspring from high nutrient parents (HL) (13.06 ± 0.21 days).

**Table 1.** Mean descriptive statistics (±s.e.) per container for offspring life-history traits, by treatment group.

treatment	*n* (containers)	female development (days)	male development (days)	survivorship (%)	female wing length (mm)
HH	8	8.32 ± 0.15	7.76 ± 0.16	60.86 ± 5.45	2.64 ± 0.02
LH	9	8.32 ± 0.09	7.54 ± 0.06	72.72 ± 3.71	2.61 ± 0.01
HL	8	13.06 ± 0.21	10.47 ± 0.17	63.31 ± 1.14	2.32 ± 0.03
LL	9	14.47 ± 0.30	11.34 ± 0.24	64.06 ± 2.19	2.29 ± 0.05

**Table 2.** MANOVA for effects of parental nutrition, offspring nutrition, and parental by offspring nutrition interaction effects on offspring development (dev) time (females, males), survivorship to adulthood and female wing length with subsequent pairwise multi-variate contrasts.

				standardized canonical coefficients			
effect	num d.f./Den d.f.	Pillai's trace	*p*-value	female dev	male dev	survivorship	female wing length
parental nutrition	4 / 26	0.47	0.0018	5.02	−0.09	0.91	−0.02
HH versus LH	4 / 11	0.41	0.17	−1.10	0.57	−0.89	0.61
HL versus LL	4 / 12	0.66	<0.0001	0.85	0.74	0.09	−0.24
offspring nutrition	4 / 26	0.97	<0.0001	4.50	0.50	0.49	−0.53
HH versus HL	4 / 10	0.97	<0.0001	4.50	0.73	0.68	−0.69
LH versus LL	4 / 13	0.97	<0.0001	4.39	0.61	0.32	−0.43
parental × offspring nutrition	4 / 26	0.31	0.0403	3.01	1.79	0.01	0.45
HH versus LL	4 / 11	0.97	<0.0001	4.68	0.66	0.76	−0.32
HL versus LH	4 / 12	0.97	<0.0001	3.72	1.35	0.05	−0.64

Multi-variate pairwise contrasts showed significant offspring nutrient effects in offspring from both high nutrient parents (*p *< 0.0001) and low nutrient parents (*p *< 0.0001). In offspring from high nutrient parents, female development time contributed most to this effect with male development time, survivorship and female size contributing similarly. Specifically, in offspring from high nutrient parents, high food females (HH) took 36% less time to develop than low nutrient females (HL). In offspring from low nutrient parents, female development time followed by male development time contributed most to this effect. Specifically, in offspring of low nutrient parents, high food females (LH) took 42.5% less time to develop than low food females (LL), and high food males (LH) took 33.5% less time to develop than low food males (LL).

Multi-variate pairwise contrasts showed significant parental by offspring nutrient effects when parents and offspring were reared in similar environments (HH versus LL, *p *< 0.0001) and when parents and offspring were reared in dissimilar environments (HL versus LH, *p *< 0.0001). When comparing high food offspring from high food parents (HH) to low food offspring from low food parents (LL), female development time followed by survivorship contributed most to this effect. Specifically, high food females from high food parents (HH) took 42.5% less time to develop than low food females from low food parents (LL). Additionally, high food offspring from high food parents (HH) had 5% reduced survivorship relative to low food offspring from low food parents (LL). When comparing high food offspring from low food parents (LH) with low food offspring from high food parents (HL), female development time followed by male development time contributed most to this effect. Specifically, female high food offspring from low food parents (LH) took 36.3% less time to develop than female low food offspring from high food parents (HL). Male high food offspring from low food parents (LH) took 28% less time to develop than male low food offspring from high food parents (HL).

### 3.2. Body infection with dengue virus

The mean dengue viral titre of the infectious blood meals was 7.2 ± 0.3 log_10_ PFUe ml^−1^, which is within the range of viraemia in humans [48]. This titre is expected to result in approximately 75% of *Ae. aegypti* blood fed with dengue to have infected bodies [49]. Of the 1900 females, a total of 266 successfully fed on the dengue infectious blood meal and were tested for infection. Of these, 92 were assayed at 24 h, 95 at 3 days and 78 at 14 days. Of the 266 females, 195 (73%) were positive for dengue in the body. Descriptive statistics for per cent infected, least square means of body titre and per cent infected with disseminated infections can be found in table 3. Contingency table analysis showed that body infection did not depend on larval nutrient treatment (P × F_1_) (*p *= 0.81, *χ*^2^ = 0.98, d.f. = 3) or day by larval nutrient treatment (P × F_1_) interaction effect (*p *= 0.27, *χ*^2^ = 7.55, d.f. = 6) for any of the treatment groups (table 4). Body infection did depend upon day of assay post-infection (*p* < 0.0001, *χ*^2^ = 89.25, d.f. = 2).

**Table 3.** Dengue virus infection and least square means for titre by treatment and day.

day	trt	no. females	% viral RNA	titre (PFUe ml^−1^)	% disseminated
1	HH	29	100 (29/29)	8975 ± 1095 (*n* = 7)	—
	LH	23	95.5 (22/23)	5585 ± 719 (*n* = 7)	—
	HL	28	96.4 (27/28)	7575 ± 1254 (*n* = 7)	—
	LL	11	100 (11/11)	7670 ± 2520 (*n* = 6)	—
3	HH	26	30.8 (8/26)	5511 ± 882 (*n* = 5)	—
	LH	24	75.0 (18/24)	12 874 ± 6938 (*n* = 8)	—
	HL	23	39.1 (9/23)	8194 ± 3879 (*n* = 8)	—
	LL	14	50.0 (7/7)	2310 ± 924 (*n* = 5)	—
14	HH	22	22.7 (5/22)	42 312 ± 12 369 (*n* = 5)	18.2 (4/22)
	LH	24	45.8 (11/24)	544 676 ± 211 083 (*n* = 7)	33.3 (8/24)
	HL	22	45.5 (10/22)	62 192 ± 29 455 (*n* = 4)	36.4 (8/22)
	LL	10	30.0 (3/10)	98 900 ± 39 448 (*n* = 3)	30.0 (3/10)

**Table 4.** Body infection by parental nutrition, parental by day treatment effects and day effect.

effect	offspring comparison	*χ*2	*p*-value	d.f.
high food F_1_				
parental treatment	HH versus LH	0.07	0.7869	1
day × parental treatment	HH versus LH	4.49	0.106	2
day	HH versus LH	56.88	<0.0001	2
low food F_1_				
parental treatment	HL versus LL	0.43	0.514	1
day × parental treatment	HL versus LL	1.70	0.428	2
day	HL versus LL	36.99	<0.0001	2

### 3.3. Viral titre and dissemination

**Figure 3.**
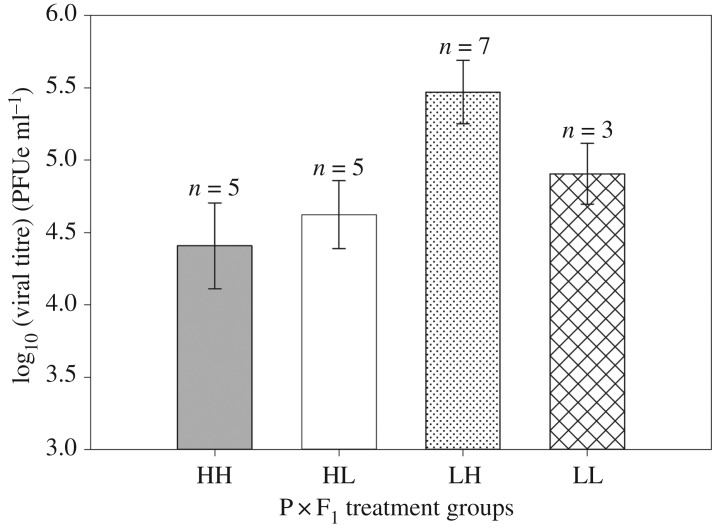
Means (±s.e.) of body dengue-1 virus titre (plaque forming unit equivalents ml^−1^) of infected offspring (14 days post-infectious blood meal) by parental by offspring nutrition treatment groups.

Mosquito viral titre was influenced by treatment (P × F_1_) (*p*= 0.0455, *F* = 2.84, d.f. = 3), day (*p* < 0.0001, *F* = 44.30, d.f. = 2) and treatment by day interaction (*p *= 0.0148, *F* = 2.92, d.f. = 6). Subsequent pairwise contrasts (electronic supplementary material, S2) found that at 24 h and 3 days, viral titres were not significantly different for treatment groups. At 14 days when offspring were reared in high nutrients, those from low nutrient parents (LH) had nearly 13 times higher viral titres (544 676 PFUe ml^−1^) compared to those with high nutrient parents (HH) (42 312 PFUe ml^−1^) (figure 3). At 14 days, offspring reared in high nutrients from low nutrient parents (LH) had 8.7 times higher viral titres (544 676 PFUe ml^−1^) compared with offspring reared in low nutrients from high nutrient parents (HL) (62 192 PFUe ml^−1^). Contingency table analysis found that larval nutrition (P × F_1_) did not significantly affect virus dissemination in offspring (*p* = 0.453, *χ*^2^ = 2.63, d.f. = 3).

## 4. Discussion

### 4.1. Life-history traits

We investigated whether parental and offspring nutrition influences offspring life-history traits (development time, survivorship and size) and susceptibility to infection with dengue. While other studies have been conducted on parental effects in mosquitoes, we are unaware of any studies on parental larval nutrition as it relates to offspring life histories and arboviral infection. Allocation of resources to life histories is influenced by environmental stress, including parental environmental stress, and underlying plasticity and constraints on these traits [50]. According to our results, offspring reared in high nutrients did not display significant differences in the life-history traits measured (male development time, female development time, female size and survivorship) regardless of parental nutrition. As holometabolous insects, mosquito larvae must allocate maternal- and larval-derived nutrients to maintenance, growth and storage [50]. When larval-derived nutrients are high, the importance of maternally derived nutrients may be lower than when larval-derived nutrients are low, especially if offspring were able to achieve the maximum growth rate for the population under the given temperature and food sources.

Offspring reared in low nutrients did display significant differences in life-history traits attributed to parental nutrition with female development time contributing the most to this effect. Specifically, females reared in low nutrients, from low nutrient parents (LL) extended the juvenile developmental period by 10% compared with females from high nutrient parents (HL). A minimum amount of nutrition is required for mosquito larvae to pupate and fully mature [51]. The differences in development time of LL females versus HL females may reflect differences in maternal egg resource allocation as a product of parent larval nutrition or reflect epigenetic imprinting. Mothers from greater nutrient larval environments may have been able to allocate more nutrients to eggs, resulting in offspring with more resources that required less from the environment to reach the threshold to develop. *Aedes aegypti* larvae display significant development time plasticity and can develop very quickly or resist starvation and develop very slowly [52]. The difference in development time due to parental larval nutrition may reflect some of this underlying plasticity. Epigenetic marking is another potential mechanism for generating parental effects in insects [9,53–55]. While survivorship contributed to the significant effect of parental larval nutrition in offspring reared in low nutrients (HL versus LL), it did so minimally and was not significantly different between these two groups. Males in low nutrients were robust to parental nutrient effects.

*Aedes aegypti* larval nutrient metabolism varies significantly by sex [51] with females requiring greater resources to pupate. This may be the reason significant differences were seen in low nutrient female offspring but not male offspring. The lower nutrient requirements of the male mosquito probably allowed males to develop more quickly regardless of parental nutrition. A study conducted on the collembolan *Orchesella cincta* showed that parental nutrition did not influence male development time; however, it did influence male weight at maturity and the production of spermatophores [56]. When considering parental effects on *Ae. aegypti* males in the future, it would be worthwhile to measure alternative life-history parameters like sperm quality.

While parental nutrition did not lead to significant differences in measured life-history traits of high nutrient offspring, survivorship did vary between offspring from low food parents (LH) and those from high food parents (HH). Offspring reared in high food, from parents reared in low food (LH) had the highest survivorship out of all four treatments. When compared with HH offspring, LH offspring had over 11% greater survivorship. The low nutrient status of LH parents may have resulted in fewer, higher quality eggs being produced allowing for greater survivorship. These results are consistent with studies on the grasshopper *Chorthippus biguttulus*, whose high nutrient parents produced superior offspring which developed faster than those from low nutrient parents [57]. Similarly, an epigenetic trade-off may exist between development and survivorship for traits that were not considered in this study.

When considering the effect of offspring nutrition, significant effects were found regardless of whether parents were reared in high or low nutrients. These effects were also primarily seen on female offspring development time with those that were reared in high nutrients developing more quickly than those reared in low nutrients, regardless of parental nutrition. High food females took 42.5% less time to develop than low food females when from high food parents and 36.4% less time to develop when from low food parents. The larger effect size from offspring nutrition relative to parental nutrition on offspring life-history traits suggests that offspring nutrition is probably more important to development than parental nutrition in this species.

### 4.2. Infection with dengue-1 virus

Feeding rates of female offspring varied by treatment and this may have biased dengue virus infection, titre and dissemination results. Body infection with dengue did not depend upon parental nutrition, day or day by parental nutrition interaction effect for any treatment groups. Offspring were equally likely to be infected with dengue regardless of treatment group. These results suggest that susceptibility to arboviral infection was not influenced by parental larval nutrition. Several studies on parental effects in arthropods have shown that parental nutrient deprivation leads to heightened resistance to pathogens [58–60], but this is inconsistent with our results. Other studies demonstrate no parental effects on offspring disease resistance [10] or parental stress leading to lower pathogen resistance [11,61]. Variation in resistance to bacterial pathogens associated with parental nutrient deprivation has been shown to vary significantly between different host genotypes in the crustacean *Daphnia magna* [59].

The mosquitoes used in this study were the F_4_ generation of a 2-year-old colony and were probably genetically similar contributing to similar infection rates across treatment groups. The proportion of mosquitoes infected was lower than some studies using *Ae. aegypti* and dengue and higher than others [45,49,62,63]. This was expected, given the importance of viral strain within serotypes [63,64], viral dose [49] and mosquito population [65] in determining susceptibility to infection. The same viral strain, viral dose and mosquito population were used in [45], yet the proportion of individuals infected in this study was much lower and may be related to differences in temperature and food between the two studies.

### 4.3. Viral titre and dissemination

Offspring treatment and offspring by day treatment significantly affected viral titres, but only after 14 days. Infection at 24 h marks the initial ingestion of the virus and its early entry into the midgut. Differences in susceptibility to infection at this point are typically seen when mosquitoes imbibe different amounts of virus [66]. As our mosquitoes were offered the same amount of virus, little to no difference in titre was expected at 24 h and this is consistent with our results. At 3 days, new virions are released from the midgut of infected mosquitoes and start infecting additional tissues [40]. During this time, Toll pathway defences against infection are active and can influence dengue titres [40]. Consequently, 3 days provide a snapshot of early infection. We did not observe any treatment differences in virus titre at 3 days, suggesting that the early immune response was not affected by parental larval nutrition.

The EIP for dengue virus in *Ae. aegypti* is generally accepted to be at 5–15 days between 25 and 28°C [39]. We used 14 days to test viral titres and to test for dissemination because it is close to the longest expected EIP and would increase our ability to detect differences among treatment groups. At 14 days, LH offspring had 13 times higher viral titres than HH offspring. The low nutrient condition of parents may have resulted in higher viral titres despite offspring being reared in high nutrients. Other studies on insects have found that offspring of low nutrient parents demonstrate reductions in the expression of innate immune markers [11,61]. Reductions in the innate immune system may explain our results; however, this is outside the scope of the present study. Life-history theory demonstrates that resources are limited and trade-offs occur in terms of how organisms invest these resources in growth, maintenance, storage and reproduction. Immune responses carry physiological costs, and trade-offs exist when allocating resources to immune function [67]. It is possible that when food conditions are limited in the parental environment, offspring have reduced immune investment as an adaptation to have additional resources for other life-history traits. It is interesting that differences in viral titres were not seen at 24 h or 3 days, but were at 14 days for HH versus LH offspring, suggesting differences late in the infection cycle. In *Ae. aegypti,* infection with dengue virus is modulated by the Toll and JAK-STAT pathways [40,41], and future studies in this system should consider these pathways and other barriers to infection.

Dissemination of the virus into leg tissues indicates the virus has escaped the midgut barrier in the mosquito. Of offspring infected at 14 days, dissemination rates were between 30 and 37% in LH, HL and LL offspring. HH offspring demonstrated dissemination rates of 18.2% of those infected. This is marginally significantly different from rates seen in LH offspring. It is possible that when offspring were reared in high nutrients, those from high nutrient parents (HH) were in better condition resulting in fewer disseminated infections relative to those from low nutrient parents (LH). It is possible that high nutrients experienced by parents and subsequent offspring may improve the efficacy of the midgut escape barrier leading to fewer disseminated infections relative to other treatment combinations. Larval nutrient deprivation has been shown to increase viral susceptibility in *Ae. aegypti* [17] as well as other vector mosquitoes [26]. Our observations marginally support the hypothesis that high-quality larval nutrition for parents and offspring led to reductions in dissemination. Viral dissemination is not a reliable indicator of transmission potential due to additional barriers to infection. However, dissemination to secondary tissue does demonstrate that the virus has bypassed the midgut escape barrier [68]. Future studies should consider parental effects on transmission.

## 5. Conclusion

Nutritional quality behaves as predicted for a stressor in which it is expected that good condition parents will produce high-quality offspring and poor condition parents will produce lower quality offspring [2,69,70]. These results suggest that parental larval nutrition is important to *Ae. aegypti* female offspring. Parental diet effects were seen on development time, infection with dengue later during the infection process, and dissemination. Carry-over effects of parental nutrition are evident in this system, and the ones measured do not appear to confer any advantages to offspring in low nutrient environments for the traits considered. Future studies should consider manipulating the types of nutrients provided, measuring offspring immune response to pathogens at earlier time points, and considering additional male traits. Additional studies are needed to assess whether parental and offspring larval diets interact to influence other parameters of vectorial capacity, an index of risk of local transmission of pathogens.

## Supplementary Material

Data File

## Supplementary Material

Pairwise Contrasts for Virus Titer in Offspring

## Supplementary Material

Dengue Virus Primers and Probes

## Supplementary Material

Statistical Codes

## Data accessibility

Our data are deposited at Dryad: http://dx.doi.org/10.5061/dryad.6c78sn1 [71].

## References

[1] Carrière Y 1994 Evolution of phenotypic variance: non-Mendelian parental influences on phenotypic and genotypic components of life-history traits in a generalist herbivore. Heredity 72, 420–430. (10.1038/hdy.1994.58)

[2] Mousseau TA, Fox CW 1998 The adaptive significance of maternal effects. Trends Ecol. Evol. 13, 403–407. (10.1016/S0169-5347(98)01472-4) 21238360

[3] Uller T 2008 Developmental plasticity and the evolution of parental effects. Trends Ecol. Evol. 23, 432–438. (10.1016/j.tree.2008.04.005) 18586350

[4] Bonduriansky R, Day T 2009 Nongenetic inheritance and its evolutionary implications. Annu. Rev. Ecol. Evol. Syst. 40, 103–125. (10.1146/annurev.ecolsys.39.110707.173441)

[5] Marshall DJ, Uller T 2007 When is a maternal effect adaptive? Oikos 116, 1957–1963. (10.1111/j.2007.0030-1299.16203.x)

[6] Vijendravarma RK, Narasimha S, Kawecki TJ 2010 Effects of parental diet on egg size and offspring traits in *Drosophila*. Biol. Lett. 6, 238–241. (10.1098/rsbl.2009.0754) 19875510 PMC2865044

[7] Rossiter MC 1996 Incidence and consequences of inherited environmental effects. Annu. Rev. Ecol. Syst. 27, 451–476. (10.1146/annurev.ecolsys.27.1.451)

[8] Grech K, Maung LA, Read AF 2007 The effect of parental rearing conditions on offspring life history in *Anopheles stephensi*. Malar. J. 6, 130–139. (10.1186/1475-2875-6-130) 17892562 PMC2034587

[9] Freitak D, Heckel DG, Vogel H 2009 Dietary-dependent transgenerational immune priming in an insect herbivore. Proc. R. Soc. B 276, 2617–2624. (10.1098/rspb.2009.0323) PMC2686660 19369263

[10] Valtonen TM, Kangassalo K, Polkki M, Rantala MJ 2012 Transgenerational effects of parental larval diet on offspring development time, adult body size and pathogen resistance in *Drosophila melanogaster*. PLoS ONE 7, e31611 (10.1371/journal.pone.0031611) 22359607 PMC3281084

[11] Triggs AM, Knell RJ 2012 Parental diet has strong transgenerational effects on offspring immunity. Funct. Ecol. 26, 1409–1417. (10.1111/j.1365-2435.2012.02051.x)

[12] Pigeault R, Garnier R, Rivero A, Gandon S 2016 Evolution of transgenerational immunity in invertebrates. Proc. R. Soc. B 283, 20161136 (10.1098/rspb.2016.1136) PMC5046895 27683366

[13] Bhatt S, Gething PW, Brady OJ, Messina JP, Farlow AW 2013 The global distribution and burden of dengue. Nature 496, 504–507. (10.1038/nature12060) 23563266 PMC3651993

[14] Murrell EG, Damal K, Lounibos LP, Juliano SA 2011 Distributions of competing container mosquitoes depend on detritus types, nutrient ratios, and food availability. Ann. Entomol. Soc. Am. 104, 688–698. (10.1603/AN10158) 22707761 PMC3375989

[15] Washburn JO 1995 Regulatory factors affecting larval mosquito populations in container and pool habitats: implications for biological control. J. Am. Mosq. Control Assoc. 11, 279–283. 7595462

[16] Telang A, Wells MA 2004 The effect of larval and adult nutrition on successful autogenous egg production by a mosquito. J. Insect Physiol. 50, 677–685. (10.1016/j.jinsphys.2004.05.001) 15234628

[17] Muturi EJ, Kim CH, Alto BW, Berenbaum MR, Schuler MA 2011 Larval environmental stress alters *Aedes aegypti* competence of Sindbis virus. Trop. Med. Int. Health 16, 955–964. (10.1111/j.1365-3156.2011.02796.x) 21564427

[18] Briegel H 1990 Metabolic relationship between female body size, reserves, and fecundity of *Aedes aegypti*. J. Insect Physiol. 36, 165–172. (10.1016/0022-1910(90)90118-Y)

[19] Farjana T, Tuno N 2013 Multiple blood feeding and host-seeking behavior in *Aedes aegypti* and *Aedes albopictus* (*Diptera: Culicidae*). J. Med. Entomol. 50, 838–846. (10.1603/ME12146) 23926783

[20] Hawley WA 1985 A high-fecundity Aedine: factors affecting egg production of the Western treehole mosquito, *Aedes sierrensis* (*Diptera: Culicidae*). J. Med. Entomol. 22, 220–225. (10.1093/jmedent/22.2.220) 3981560

[21] Takken W, Klowden MJ, Chambers GM 1998 Effect of body size on host seeking and blood meal utilization in *Anopheles gambiae* sensu stricto (Diptera: Culicidae): the disadvantage of being small. J. Med. Entomol. 35, 639–645. (10.1093/jmedent/35.5.639) 9775585

[22] Klowden MJ, Lea AO 1978 Blood meal size as a factor affecting continued host-seeking by *Aedes aegypti* (L.). Am. J. Trop. Med. Hyg. 27, 827–831. (10.4269/ajtmh.1978.27.827) 686250

[23] Lyimo EO, Takken W 1993 Effects of adult body size on fecundity and the pre-gravid rate of *Anopheles gambiae* females in Tanzania. Med. Vet. Entomol. 7, 328–332. (10.1111/j.1365-2915.1993.tb00700.x) 8268486

[24] Scott TW, Takken W 2012 Feeding strategies of anthropophilic mosquitoes result in increased risk of pathogen transmission. Trends Parasitol. 28, 114–121. (10.1016/j.pt.2012.01.001) 22300806

[25] Zhang S, He G, Xu L, Lin Q, Zhang S 1993 Effects of larval nutrition on susceptibility of *Aedes albopictus* to dengue 2 virus. Arbovirus Res Aust. 6, 44–48.

[26] Grimstad PR, Walker ED 1991 *Aedes triseriatus* (*Diptera: Culicidae*) and La Crosse virus. IV. Nutritional deprivation of larvae affects adult barriers to infection and transmission. J. Med. Entomol. 28, 378–386. (10.1093/jmedent/28.3.378) 1875364

[27] Takahashi M 1976 The effects of environmental and physiological conditions of *Culex tritaeniorhynchus* on the pattern of transmission of Japanese encephalitis virus. J. Med. Entomol. 13, 275–284. (10.1093/jmedent/13.3.275) 1011230

[28] Nasci RS, Mitchell CJ 1993 Larval diet, adult size and susceptibility of *Aedes aegypti* (*Diptera: Culicidae*) to infection with Ross River virus. J. Med. Entomol. 31, 123–126. (10.1093/jmedent/31.1.123) 8158614

[29] Dodson BL, Kramer LD, Rasgon JL 2011 Larval nutritional stress does not affect vector competence for West Nile virus (WNV) in *Culex tarsalis*. Vector Borne Zoonotic Dis. 11, 1493–1497. (10.1089/vbz.2011.0662) 21867417 PMC3216062

[30] Telang A, Qayum AA, Parker A, Sacchetta BR, Byrnes GR 2012 Larval nutritional stress affects vector immune traits in adult yellow fever mosquito *Aedes aegypti* (*Stegomyia aegypti*). Med. Vet. Entomol. 26, 271–281. (10.1111/j.1365-2915.2011.00993.x) 22112201

[31] Lorenz LM, Koella JC 2011 Maternal environment shapes the life history and susceptibility to malaria of *Anopheles gambiae* mosquitoes. Malar. J. 10, 382 (10.1186/1475-2875-10-382) 22188602 PMC3269443

[32] Otti O, Sadd BM 2008 Parental guidance? Transgenerational influences on offspring life history in mosquitoes. Trends Parasitol. 24, 197–199. (10.1016/j.pt.2008.02.004) 18406210

[33] Centers for Disease Control and Prevention (CDC). 2010 Locally acquired dengue—Key West, Florida, 2009–2010. Morb. Mortal. Wkly. Rep. 59, 577–581. 20489680

[34] Mousseau TA 2000 Intra- and interpopulational genetic variation. In Adaptive genetic variation in the wild (eds Mousseau TA, Sinervo B, Endler J), pp. 219–250. New York, NY: Oxford University Press.

[35] Daugherty MP, Alto BW, Juliano SA 2000 Invertebrate carcasses as a resource for competing *Aedes albopictus* and *Aedes aegypti* (Diptera: Culicidae). J. Med. Entomol. 37, 364–372. (10.1093/jmedent/37.3.364) 15535579 PMC2579927

[36] Alto BW, Lounibos LP, Higgs S, Juliano SA 2005 Larval competition differentially affects arbovirus infection in *Aedes* mosquitoes. Ecology 86, 3279–3288. (10.1890/05-0209) 19096729 PMC2605070

[37] Alto BW, Bettinardi D 2013 Temperature and dengue virus infection in mosquitoes: independent effects on the immature and adult stages. Am. J. Trop. Med. Hyg. 88, 497–505. (10.4269/ajtmh.12-0421) 23382163 PMC3592531

[38] Alto BW, Lounibos LP, Mores CN, Reiskind MH 2008 Larval competition alters susceptibility of adult *Aedes* mosquitoes to dengue infection. Proc. R. Soc. B 275, 463–471. (10.1098/rspb.2007.1497) PMC2289994 18077250

[39] Tjaden NB, Thomas SM, Fischer D, Beierkuhnlein C 2013 Extrinsic incubation period of dengue: knowledge, backlog, and application of temperature dependence. PLoS Negl. Trop. Dis. 7, e2207 (10.1371/journal.pntd.0002207) 23826399 PMC3694834

[40] Ramirez JL, Dimopoulos G 2010 The Toll immune signaling pathway control conserved anti-dengue defenses across diverse *Aedes aegypti* strains and against multiple dengue virus serotypes. Dev. Comp. Immunol. 34, 625–629. (10.1016/j.dci.2010.01.006) 20079370 PMC2917001

[41] Xi Z, Ramirez JL, Dimopoulos G 2008 The *Aedes aegypti* Toll pathway controls dengue virus infection. PLoS Pathog. 4, e1000098 (10.1371/journal.ppat.1000098) 18604274 PMC2435278

[42] Armbruster P, Hutchinson R 2002 Pupal mass and wing length as indicators of fecundity in *Aedes albopictus* and *Aedes* g*eniculatus* (*Diptera: Culicidae*). J. Med. Entomol. 39, 699–704. (10.1603/0022-2585-39.4.699) 12144308

[43] Turell MJ, Gargan TP II, Bailey CL 1984 Replication and dissemination of Rift Valley fever virus in *Culex pipiens*. Am. J. Trop. Med. Hyg. 22, 176–181. (10.4269/ajtmh.1984.33.176) 6696176

[44] Callahan JD et al. 2001 Development and evaluation of serotype and group-specific fluorogenic reverse transcriptase PCR (TaqMan) assays for dengue virus. J. Clin. Microbiol. 39, 4119–4124. (10.1128/JCM.39.11.4119-4124.2001) 11682539 PMC88496

[45] Buckner EA, Alto BW, Lounibos LP 2013 Vertical transmission of Key West dengue-1 virus by *Aedes aegypti* and *Aedes albopictus* (*Diptera: Culicidae*) mosquitoes from Florida. J. Med. Entomol. 50, 1291–1297. (10.1603/ME13047) 24843934 PMC4031614

[46] Scheiner SM 2001 MANOVA: multiple response variables and multispecies interactions. In Design and analysis of ecological experiments (eds Scheiner SM, Gurevitch J), pp. 99–115. Oxford, UK: Oxford University Press.

[47] Rice WR 1989 Analyzing tables of statistical tests. Evolution 43, 223–225. (10.1111/j.1558-5646.1989.tb04220.x) 28568501

[48] Stramer SL et al. 2012 Dengue viremia in blood donors identified by RNA and detection of dengue transfusion transmission during the 2007 dengue outbreak in Puerto Rico. Transfusion 52, 1657–1666. (10.1111/j.1537-2995.2012.03566.x) 22339201

[49] Nguyet NM et al. 2013 Host and viral features of human dengue cases shape the population of infected and infectious *Aedes aegypti* mosquitoes. Proc. Natl Acad. Sci. USA 110, 9072–9077. (10.1073/pnas.1303395110) 23674683 PMC3670336

[50] Boggs CL 2009 Understanding insect life histories and senescence through a resource allocation lens. Funct. Ecol. 23, 27–37. (10.1111/j.1365-2435.2009.01527.x)

[51] Chambers GM, Klowden MJ 1990 Correlation of nutritional reserves with a critical weight for pupation in larval *Aedes aegypti* mosquitoes. J. Am. Mosq. Control Assoc. 6, 394–399. 2230767

[52] Barrera R, Medialdea V 1996 Development time and resistance to starvation of mosquito larvae. J. Nat. Hist. 30, 447–458. (10.1080/00222939600770231)

[53] Bongiorni S, Cintio O, Prantera G 1999 The relationship between DNA methylation and chromosome imprinting in the Coccid *Planococcus citri*. Genetics 151, 1471–1478. 10101170 10.1093/genetics/151.4.1471 PMC1460555

[54] Bonduriansky R, Head MH 2007 Maternal and paternal condition effects on offspring phenotype in *Telostylinus angusticollis* (Diptera: Neriidae). J. Evol. Biol. 20, 2379–2388. (10.1111/j.1420-9101.2007.01419.x) 17956399

[55] MacDonald WA 2012 Epigenetic mechanisms of genomic imprinting: common themes in the regulation of imprinted regions in mammals, plants, and insects. Genet. Res. Int. 2012, 1–17. (10.1155/2012/585024) PMC3335465 22567394

[56] Zizzari ZV, van Straalen NM, Ellers J 2016 Transgenerational effects of nutrition are different for sons and daughters. J. Evol. Biol. 29, 1317–1327. (10.1111/jeb.12872) 27018780

[57] Franzke A, Reinhold K 2012 Transgenerational effects of diet environment on life-history and acoustic signals of a grasshopper. Behav. Ecol. 24, 734–739. (10.1093/beheco/ars205)

[58] Ben-Ami F, Ebert D, Regoes RR 2010 Pathogen dose infectivity curves as a method to analyze the distribution of host susceptibility: a quantitative assessment of maternal effects after food stress and pathogen exposure. Am. Nat. 175, 106–115. (10.1086/648672) 19911987

[59] Stjernman M, Little TJ 2011 Genetic variation for maternal effects on parasite susceptibility. J. Evol. Biol. 24, 2357–2363. (10.1111/j.1420-9101.2011.02363.x) 21848987

[60] Shikano I, Oak MC, Halpert-Scanderberg O, Cory JS 2015 Trade-offs between transgenerational transfer of nutritional stress tolerance and immune priming. Funct. Ecol. 29, 1156–1164. (10.1111/1365-2435.12422)

[61] Saastamoinen M, Hirai N, van Nouhuys S 2013 Direct and trans-generational responses to food deprivation during development in the Glanville fritillary butterfly. Oecologia 171, 93–104. (10.1007/s00442-012-2412-y) 22814878

[62] Richards SL, Anderson SL, Alto BW 2012 Vector competence of *Aedes aegypti* and *Aedes albopictus* (Diptera: Culicidae) for dengue virus in the Florida Keys. J. Med. Entomol. 49, 942–946. (10.1603/ME11293) 22897056

[63] Alto BW, Smartt CT, Shin D, Bettinardi D, Malicoate J, Anderson SL, Richards SL 2014 Susceptibility of Florida *Aedes aegypti* and *Aedes albopictus* to dengue viruses from Puerto Rico. J. Vector Ecol. 39, 406–413. (10.1111/jvec.12116) 25424270

[64] Lambrechts L, Chevillon C, Albright RG, Thaisomboonsuk B, Richardson JH, Jarman RG, Scott TW 2009 Genetic specificity and potential for local adaptation between dengue viruses and mosquito vectors. BMC Evol. Biol. 9, 160 (10.1186/1471-2148-9-160) 19589156 PMC2714696

[65] Bennett KE, Olson KE, Munoz M de L, Fernandez-Salas I, Farfan-Ale JA, Higgs S, Black WC IV, Beaty BJ 2002 Variation in vector competence for dengue 2 virus among 24 collections of *Aedes aegypti* from Mexico and the United States. Am. J. Trop. Med. Hyg. 67, 85–92. (10.4269/ajtmh.2002.67.85) 12363070

[66] Gubler DJ, Nalim S, Tan R, Saipan H, Sulianti Saroso J 1979 Variation in susceptibility to oral infection with dengue viruses among geographic strains of *Aedes aegypti*. Am. J. Trop. Med. Hyg. 28, 1045–1052. (10.4269/ajtmh.1979.28.1045) 507282

[67] Rauw WM 2012 Immune response from a resource allocation perspective. Front. Genet. 3, 267 (10.3389/fgene.2012.00267) 23413205 PMC3571735

[68] Chamberlain RW, Sudia WD 1961 Mechanism of transmission of viruses by mosquitoes. Annu. Rev. Entomol. 6, 371–390. (10.1146/annurev.en.06.010161.002103) 13692218

[69] Kyneb A, Toft S 2006 Effects of maternal diet quality on offspring performance in the rove beetle *Tachyporus hypnorum*. Ecol. Entomol. 31, 322–330. (10.1111/j.1365-2311.2006.00775.x)

[70] Jones TM, Widemo F 2005 Survival and reproduction when food is scarce: implications for a lekking Hawaiian *Drosophila*. Ecol. Entomol. 30, 397–405. (10.1111/j.0307-6946.2005.00705.x)

[71] Zirbel K, Eastmond B, Alto BW 2018 Data from: Parental and offspring larval diets interact to influence life history traits and infection with dengue virus in *Aedes aegypti* Dryad Digital Repository (10.5061/dryad.6c78sn1) PMC6083674 30109101

