# Supplementary material for: Parental and offspring larval diets interact to influence life-history traits and infection with dengue virus in Aedes aegypti
Source: R Soc Open Sci. 2018 Jul 18;5(7):180539. doi: 10.1098/rsos.180539 (PMC6083674; doi:10.1098/rsos.180539)
Supplement: Data File [file rsos180539supp1.docx]

Table S1. Dengue-1 serotype specific primers and probes designed by [Callahan et al. 2001](#Callahan_et_al_2001).

| Primer/Probe | Sequence (5’—3’) | Genomic Region | GenBank # |
| --- | --- | --- | --- |
| Forward Primer | GAC ACC ACA CCC TTT GGA CAA | NS5 (8586-8606) | M87512 |
| Reverse Primer | CAC CTG GCT GTC ACC TCC AT | NS5 (8692-8673) |  |
| Probe | AGA GGG TGT TTA AAG AGA AAG TTG ACA CGC G | NS5 (8606-8638) |  |
