# Supplementary material for: Parental and offspring larval diets interact to influence life-history traits and infection with dengue virus in Aedes aegypti
Source: R Soc Open Sci. 2018 Jul 18;5(7):180539. doi: 10.1098/rsos.180539 (PMC6083674; doi:10.1098/rsos.180539)
Supplement: Pairwise Contrasts for Virus Titer in Offspring [file rsos180539supp2.docx]

Table S2. Pairwise contrasts for Log10 virus titer in offspring by treatment (trt) and day adjusted using the Bonferroni method.

| Trt 1 | Trt 2 | Day | Estimate | S.E. | d.f. | T Value | Pr>\|t\| | Critical *p* |
| --- | --- | --- | --- | --- | --- | --- | --- | --- |
| HH | HL | 1 | 0.2003 | 0.5636 | 58 | 0.36 | 0.7236 | 0.0125 |
| HH | LH | 1 | 0.4800 | 0.5636 | 58 | 0.85 | 0.3979 | 0.0062 |
| HH | LL | 1 | 0.3708 | 0.5866 | 58 | 0.63 | 0.5298 | 0.0071 |
| HL | LH | 1 | 0.2797 | 0.5636 | 58 | 0.50 | 0.6216 | 0.01 |
| HL | LL | 1 | 0.1705 | 0.5866 | 58 | 0.29 | 0.7724 | 0.0166 |
| LH | LL | 1 | -0.1092 | 0.5866 | 58 | -0.19 | 0.8529 | 0.025 |
| HH | HL | 3 | 0.6741 | 0.6384 | 58 | 1.06 | 0.2954 | 0.0045 |
| HH | LH | 3 | -0.08353 | 0.6011 | 58 | -0.14 | 0.8900 | 0.05 |
| HH | LL | 3 | 1.2698 | 0.6668 | 58 | 1.90 | 0.0618 | 0.0033 |
| HL | LH | 3 | -0.7576 | 0.5694 | 58 | -1.33 | 0.1886 | 0.0041 |
| HL | LL | 3 | 0.5957 | 0.6384 | 58 | 0.93 | 0.3547 | 0.0055 |
| LH | LL | 3 | 1.3533 | 0.6011 | 58 | 2.25 | 0.0282 | 0.0031 |
| HH | HL | 14 | -0.3770 | 0.7073 | 58 | -0.53 | 0.5960 | 0.0083 |
| HH | LH | 14 | -2.4464 | 0.6174 | 58 | -3.96 | 0.0002* | 0.0027 |
| HH | LL | 14 | -1.1468 | 0.7700 | 58 | -1.49 | 0.1418 | 0.0038 |
| HL | LH | 14 | -2.0694 | 0.6609 | 58 | -3.13 | 0.0027* | 0.0029 |
| HL | LL | 14 | -0.7697 | 0.8053 | 58 | -0.96 | 0.3431 | 0.005 |
| LH | LL | 14 | 1.2996 | 0.7276 | 58 | 1.79 | 0.0793 | 0.0035 |

*Denotes significant p-value after Bonferroni correction
