# Supplementary material for: Parental and offspring larval diets interact to influence life-history traits and infection with dengue virus in Aedes aegypti
Source: R Soc Open Sci. 2018 Jul 18;5(7):180539. doi: 10.1098/rsos.180539 (PMC6083674; doi:10.1098/rsos.180539)
Supplement: Statistical Codes [file rsos180539supp4.docx]

STATISTICS SAS CODES

Generalized Linear Mixed Models

**data**;

input;

Lines;;

**PROC** **glimmix** data= plots=pearsonpanel; *plots pearson residuals;

title "";

class;

model = /ddfm=kr;

random _residual_/group=day;

lsmeans /lines adjust=tukey;

**run**;

Multivariate Analysis of Variance

**data**;

input;

cards;;

**proc** **glm** data=;

class;

model /ss3;

lsmeans /stderr pdiff adjust=tukey;

manova h=_all_ /canonical;

**run**;

Maximum Likelihood Categorical Analyses of Contingency Tables

**data**;

input @@;

datalines;;

**proc genmod**;

weight;

class;

model = /type3 scale=pearson dist=bin;

lsmeans /adjust=tukey lines;

**run;**
